# Genetic Diversity of Astrovirus in Children From a Birth Cohort in Nepal

**DOI:** 10.3389/fmicb.2020.588707

**Published:** 2021-02-05

**Authors:** Sanjaya Kumar Shrestha, Jasmin Shrestha, Ashild K. Andreassen, Tor A. Strand, Susanne Dudman, Jennifer L. Dembinski

**Affiliations:** ^1^Center for International Health, University of Bergen, Bergen, Norway; ^2^Walter Reed/Armed Forces Research Institute of Medical Sciences Research Unit Nepal, Kathmandu, Nepal; ^3^Department of Virology, Norwegian Institute of Public Health, Oslo, Norway; ^4^Department of Research, Innlandet Hospital Trust, Lillehammer, Norway; ^5^Institute of Clinical Medicine, University of Oslo, Oslo, Norway

**Keywords:** astrovirus, Nepal, genetic diversity, diarrhea, children

## Abstract

**Objective:** This study describes the types of Human astroviruses detected in stool samples collected from a birth cohort of children in Nepal.

**Methods:** Using a commercial kit (ProSpecT), a total of 5,224 diarrheal and non-diarrheal stool samples were screened for Human astrovirus by ELISA. RT-PCR was performed on ELISA positive samples (2.8%) for further confirmation. The primary RT-PCR assay used targets the ORF2 region and detects human astrovirus type 1–8. Samples that were negative in this assay were further analyzed using primers that target the ORF1b region of human astrovirus which detect both classical type (HAstV 1–8) and novel types (MLB1–5, VA 1–5). PCR positive samples were analyzed by Sanger sequencing to determine the genotype.

**Results:** A total of 148 available ELISA positive stool samples were analyzed by RT-PCR and further genotyped. RT-PCR analysis of these samples using the ORF2 and ORF1b assay revealed that 124 (84%) were positive for classical human types (HAstV 1–8). Seven different classical HAstV genotypes based on ORF2 and ORF1a were identified (HAstV 1- HAstV 8) with the greatest prevalence of HAstV 5 genotype (42.2%), followed by HAstV 1 (34.7%), HAstV 2 and HAstV 8 (7.4%), HAstV 4 (4.1%), HAstV 3 (3.3%), and HAstV 6 (0.8%). Non-classical types were not detected in our study.

**Conclusion:** A high diversity of circulating Astrovirus strains were detected in young children, both with and without symptoms of gastroenteritis. HAstV 5 and HAstV 1 were the most common genotypes in young children in Nepal.

## Introduction

Diarrheal disease is a major public health problem globally. It is estimated 525,000 deaths occur annually in children younger than 5 years due to diarrhea, even though diarrheal disease is preventable and treatable (World Health Organization, 2017).

The etiological agents for acute diarrhea include different pathogens. Even though diarrhea can be caused by different bacteria and protozoa, 75% or more of cases can be caused by viruses (Mohammad et al., 2020) which includes, rotavirus, norovirus, and human astrovirus (HAstV), enteric adenovirus, and Sapporo virus.

Astroviruses are known to infect variety of mammalian species (Koci and Schultz-Cherry, 2002; Chu et al., 2008; Toffan et al., 2009) and are transmitted by fecal oral route causing gastroenteritis. Viruses within the family “Astroviridae,” are non-lipid enveloped, single-stranded RNA viruses with 6.4–7.3 kb genome size. The genome is coded for three open reading frames: ORF1a, 1b, and 2 which encode a serine protease, an RNA-dependent RNA polymerase (RdRp) and a capsid precursor protein, respectively (Mendez and Arias, 2007; Kapoor et al., 2009).

Human astrovirus were initially identified and classified into eight serotypes (Kjeldsberg, 1994; Kazushi et al., 2000) and these are estimated to cause about 10% of sporadic diarrhea cases (Kirkwood, 2005). HAstV type 1 is the most prevalent, whereas HAstV 7 and HAstV 8 are rare (Wang et al., 2001). A highly divergent types astrovirus HAstV-MLB was detected in a child with diarrhea in Australia (Finkbeiner et al., 2008, 2009) and India (Finkbeiner et al., 2009). Verma et al. (2010) described “sporadic cases of AGE in children due to dual genotype HAstV strains from Western India in 2010.” Recently, different variant and inter-genotype recombinant HAstV strains have been reported from Kolkata to cause gastroenteritis in infants, children, and adults (Pativada et al., 2011). Two novel astroviruses VA4 and MLB3 have been identified from pediatric stool samples collected from Nepal and India (Jiang et al., 2013). Many studies reported diarrhea caused by various viruses but less information is available about human astrovirus causing gastroenteritis in <5 years of children in Nepal. This study aims to find the prevalence, age distribution, seasonality, and genetic diversity of human astrovirus in a longitudinal birth cohort of children followed for 3 years in Bhaktapur, Nepal.

## Methods

### Study Population and Sample Collection

The stool samples used in this study were collected from June 2010 through February 2015 under the protocol titled “Etiology, Risk Factors, and Interactions of Enteric Infection and Malnutrition and the Consequences for Child Health and Development in Nepal (MAL-ED)” and archived. This was part of a multicenter study carried out simultaneously at 8 countries including Bangladesh, Brazil, India, Pakistan, Peru, South Africa, Tanzania, and Nepal. The study population and procedures for sample collection done in Nepal has been described in previously published paper (Investigators, 2014; Shrestha et al., 2014).

Different enteric pathogens were identified from stool samples following standard procedures in the MAL-ED study (Houpt et al., 2014). A total of 5,224 stool samples, 1,160 diarrheal stool samples and 4,064 non-diarrheal monthly stool samples were collected from 240 children from birth up to age of 36 months and were screened for enteric viruses Astrovirus, Rotavirus and Adenovirus using commercial ProSpect T enzyme linked immunosorbent assay (ELISA) (Remel, obtained from Fisher Scientific, Pittsburgh, PA), according to manufacturers' instructions. Preserved stool samples that were screened positive for Astrovirus were selected for strain identification.

### Nucleic Acid Extraction and Real-Time PCR

A 10% suspension of fecal samples were prepared in 0.85% NaCl and centrifuged at 4,000 g for 20 min. One hundred and forty microliter of the supernatant was used as starting material for viral nucleic acid extraction by the QIAamp Viral RNA kit (QIAGEN, Hilden, Germany). Astrovirus detection was performed using reverse transcription polymerase chain reaction (RT-PCR) for astrovirus detection, was performed as described by Noel et al. (1995). Screenings of classical human astrovirus were done by specific primers MON269 (forward 5′- CAACTCAGGAAACAGGGTGT-3′) and MON270 (reverse 5′-TCAGATGCATTGTCATTGGT-3′), which amplify a 449 bp region located in ORF2. QIAGEN One-Step RT-PCR kit (QIAGEN, Hilden, Germany) following cycling conditions: 30 min RT step, 94°C hold for 10 min, followed by 40 cycles of 94°C for 30 s, 56°C for 30 s, and 72°C for 50 s was used in the reaction. The final products (10 ul) were visualized after electrophoresis on a 1.5% agarose gel with SYBR stain. If the results from the specific screening primers were negative, the sample was retested using the consensus primers SF0073 (5′-GATTGGACTCGATTTGATGG-3′) and SF0076 (5′-CTGGCTTAACCCACATTCC-3′) targeting highly conserved regions in the ORF 1b (RNA polymerase) of both classical as well as novel types (MLB and VA) astroviruses.

### Astrovirus Typing

Molecular characterization of HAstV strains was performed using PCR products which were sequenced using the same pair of primers used for the amplification reactions, MON269 and MON270 or SF0073 and SF0076 primers in both directions using the ABI Prism 3,500 Genetic Analyzer and Big Dye Terminator Cycle Sequencing Kit v.3.1 (Applied Biosystems, Foster City,CA).

### Data Analysis

All analysis was done using excel. The odds ratio and corresponding 95% confident intervals were calculated for each age group in diarrhea vs. normal stools. All tests were considered to be statistically significant at a *p* < 0.05.

### Phylogenetic Analysis

The sequences were analyzed and aligned by Sequencher version 5.4.6 (GeneCodes) and BioEdit version 7.2.5 (Hall, 1999) programs. Similarity, NCBI basic local alignment search tool (BLAST) was used to compare sequence data against the library Genebank database. Reference sequences available for the same genomic region (ORF1b or ORF2) and size were selected considering each genetic type of HAstV for phylogenetic analysis. All sample data has been deposited into GenBank (accession numbers MW340996–MW341109). Trees were created by MEGA software version 6. The neighbor-joining method and bootstrap analysis with 100 reads were used in the analysis.

### Ethical Approval

The study was approved by ethical committee of the Nepal Health Research Council as national IRB of Nepal (Reg. no. 222/2015) and the Norwegian regional committee for medical and health research ethics (REK-sør-øst B 42335).

## Results

### Prevalence and Coinfection

**Figure 1:**
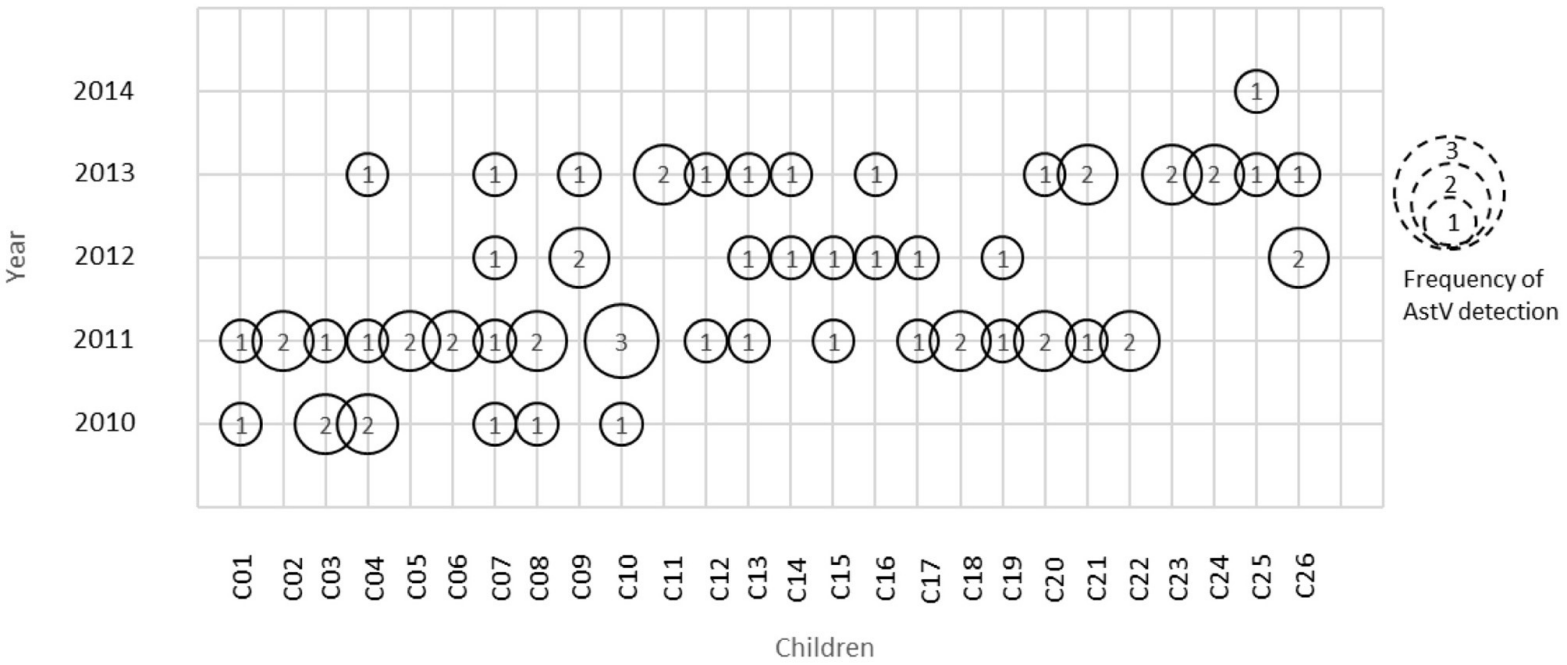
Astrovirus detection in children at different time period. Numbers inside circle represents frequency of astrovirus detection in that particular year.

A total of 149 stool samples from 110 children were screened positive for Astrovirus by ELISA. Out of the 149 positive stool samples, 55/1,160 (4.7%) were from diarrheal and 94/4,064 (2.3%) were from non-diarrheal stool. Children enrolled in this study were classified in different age groups 0–12, 13–25, and 26–36 months (Table 1). Diarrhea due to HAstV, was seen in all age groups with odd ratio (OR) more than 1 but was significant only in age groups 13–25 months (*p* < 0.00) and 26–36 months (*p* < 0.039). Mono-infection with only astrovirus was found in 57/149 (38%) samples, whereas mixed infection with one or more target pathogens were found in 92/149 (62%) (Table 2). Coinfection with bacteria was detected in both diarrheal as well as non-diarrheal stools but was seen more frequently in asymptomatic stool. In contrast to this, viral coinfection was seen more in stool with diarrhea. Astrovirus was mostly seen as a coinfection with bacteria as compared to virus and parasites. Astrovirus was detected together with *E. coli*, Campylobacter and *Shigella* spp. and also found coinfecting with other enteric viruses like Norovirus GII and Rotavirus. Astrovirus was detected multiple times in 26 children in 36 months of age (Figure 1). Average stool collection including diarrheal and non-diarrheal for those 26 children is 24 times except for one child whose stool was collected only two times. Out of 26 children reoccurrence of astrovirus detection was seen in 18 children, 4 children showed prolong detection in subsequent stool samples and both prolonged astrovirus detection and reoccurrence was noted in 4 children.

**Table 1:** Astrovirus prevalence in different age groups.

	**Diarrhea (Case) *N =* 1,160**		**Monthly (Control) *N =* 4,064**				
**Age (Month)**	**Positive (%)**	**Negative (%)**	**Positive (%)**	**Negative (%)**	**OR**	**95% CI**	***P*-value**
0–12	22 (3.78)	560 (96.2)	70 (2.56)	2,632 (97.4)	1.48	0.91–2.41	0.117
13–25	27 (6.04)	420 (93.95)	17 (1.91)	870 (98)	3.29	1.77–6.10	**0.000**
26–38	6 (4.58)	125 (95.41)	7 (1.47)	468 (98.5)	3.21	1.06–9.72	**0.039**

*The bold values describes significance*.

**Table 2:** Astrovirus co-infections with bacteria, virus, and parasites.

**Organisms identified**	**Total numbers**
Astrovirus	57
2 Pathogens	
Astrovirus, Norovirus GII	3
Astrovirus, Rotavirus	2
Astrovirus, *Campylobater*	16
Astrovirus, *Shigella dysenteriae*	2
Astrovirus, *E. coli*	37
Astrovirus, *E. histolytica*	1
Astrovirus, Giardia	8
3 Pathogens	
Astrovirus, Adenovirus, *E. coli*	1
Astrovirus, Norovirus GII, *Campylobacter*	1
Astrovirus, Norovirus GII, *E. coli*	1
Astrovirus, *Campylobater, E. coli*	7
Astrovirus, *Shigella flexneri, E. coli*	1
Astrovirus, *E. coli*, Giardia	1
Astrovirus, *Campylobacter*, Giardia	7
Astrovirus, *Campylobater, E. Histolytica*	1
4 Pathogens	
Astrovirus, *E. coli*, Giardia, Norovirus GII	1
Astrovirus, *Campylobacter*, Norovirus GII, *E. Histolytica*	1
Astrovirus, *Campylobacter*, Cryptosporidium, Giardia	1

### Confirmatory Astrovirus PCR Test

**Figure 2:**
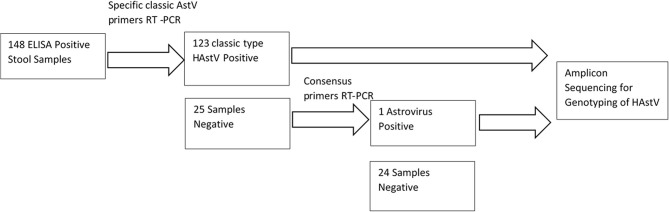
Schematic diagram of RT-PCR screening of astrovirus for sequencing.

One hundred and forty eight out of the 149 astrovirus positive samples available were tested using specific primers for detection of classical Astrovirus type 1–8. Only 123 (83%) samples were positive for classic type astrovirus type (1–8). Negative samples (*N* = 25), were screened using the second set of primers targeting the highly conserved region of both classical and novel MLB and VA Astrovirus, and showed one positive sample (Figure 2). This positive sample will be further characterized by sequencing method. In total 16% (24/148) of samples were negative and 124/148 were positive (84%) for HAstV with PCR.

### Genotypes and Seasonal Distribution of Astrovirus Infection

**Figure 3:**
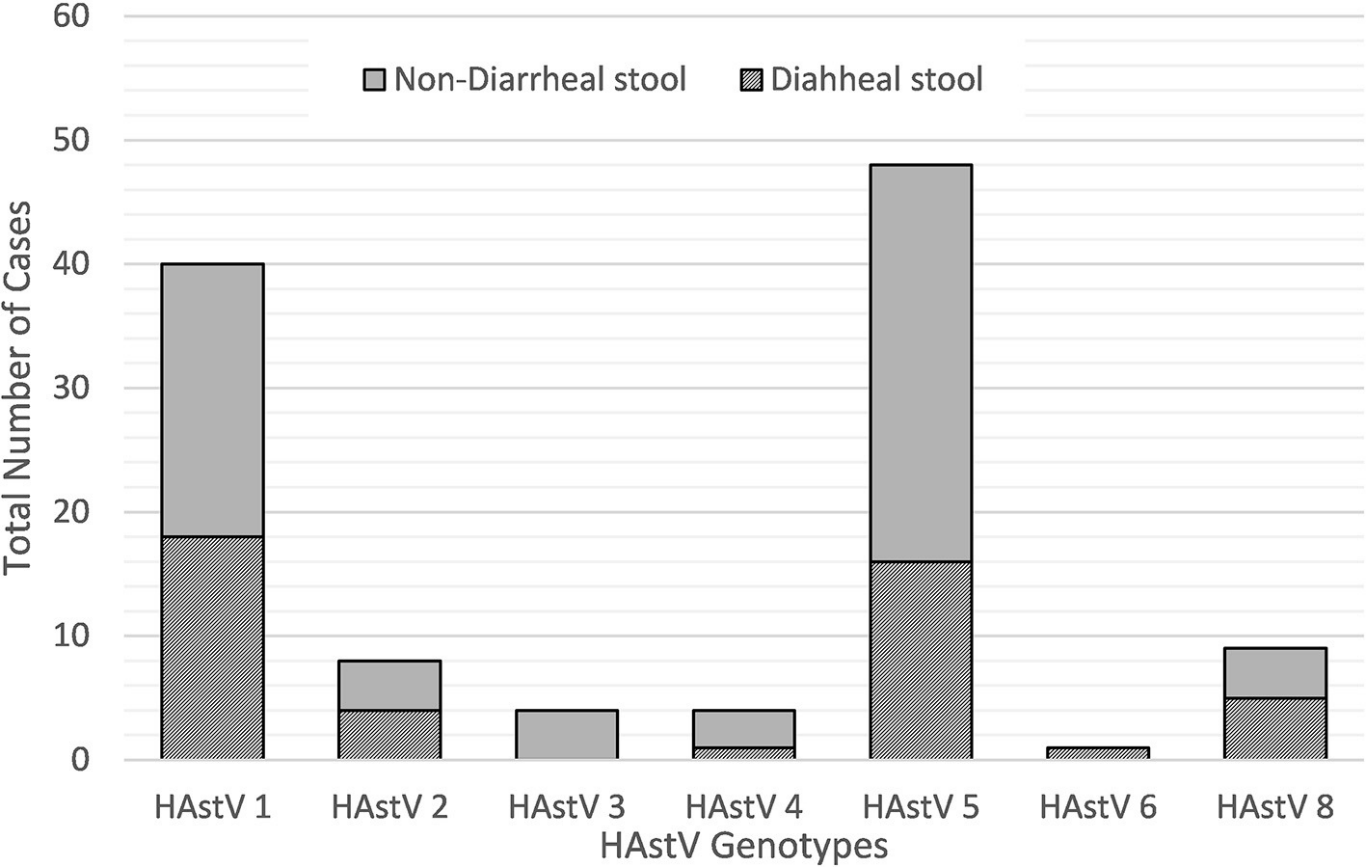
Astrovirus genotypes identified by sequencing in different stool types.

During the 2010–2015 study period, a wide variety of astrovirus genotypes were circulating in this child population as identified through sequencing. Of the 124 PCR positive samples, 121 were successfully sequenced with 114 samples generating high quality data to be included in this analysis. Overall, seven different genotypes of classic type human astrovirus (HAstV 1, HAstV 2, HAstV 3, HAstV 4, HAstV 5, HAstV 6, and HAstV 8) were identified as shown in Figure 3. HAstV 5 genotype (51/121, 42.2%) dominated among other HAstV, followed by HAstV 1 (42/121, 34.7%), HAstV 2 and HAstV 8 (9/121, 7.4%), HAstV 4 (5/121, 4.1%), HAstV 3 (4/121, 3.3%), and HAstV 6 (1/121, 0.8%). The distribution of genotypes varied across the study period. HAstV 5 dominated in 2011 and 2013 whereas in 2012 HAstV 1 was the predominant genotype. HAstv 1 was detected throughout all years of the study. Similarly, HAstV 5, and HAstV 8 were circulating in 2010–2013, while HAstV 6 was only detected in 2013.

**Figure 4:**
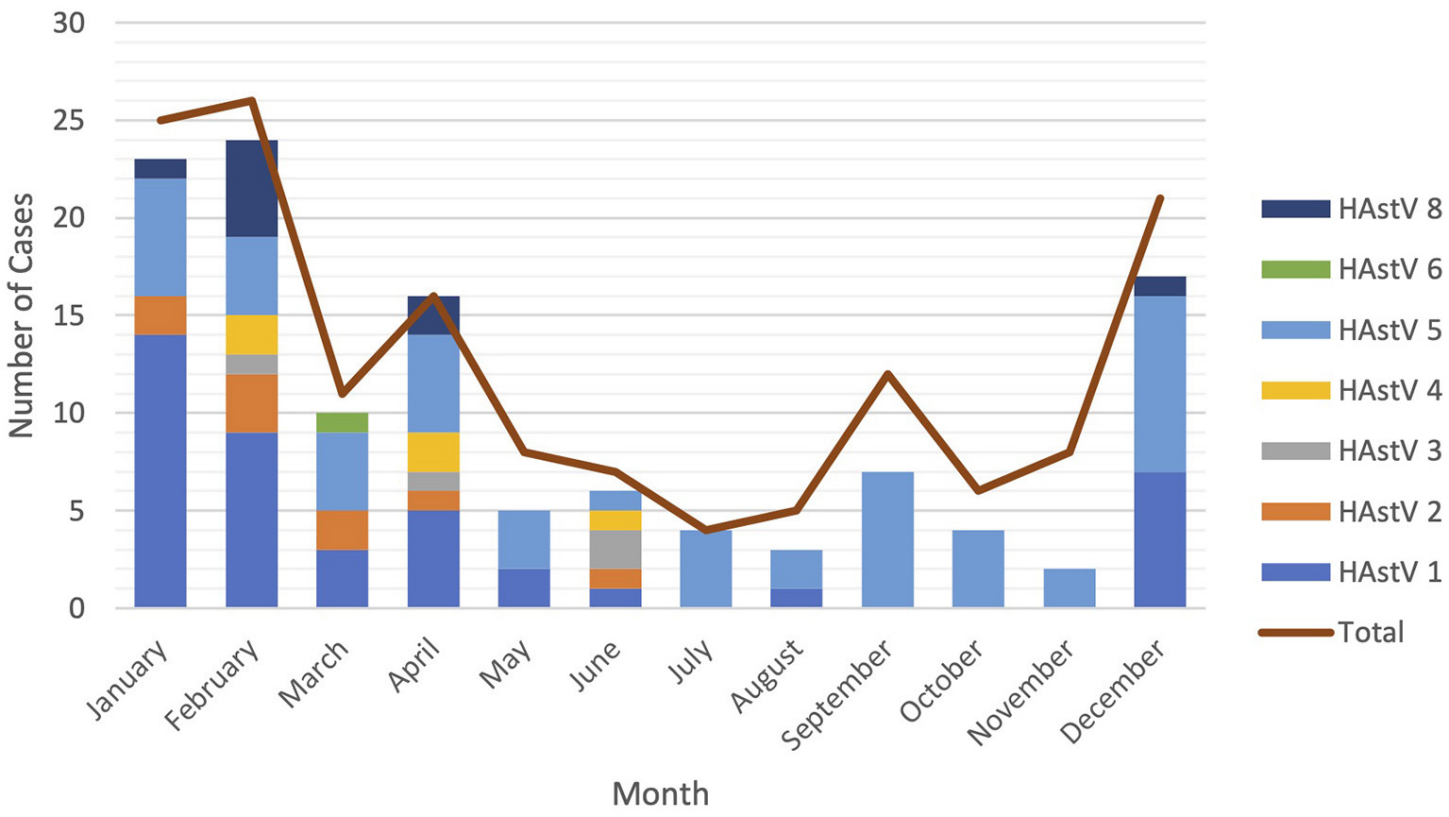
Seasonal distribution of astrovirus genotypes. Different color bars represents different genotypes of astrovirus. Red line is total cases of astrovirus detected monthly.

**Figure 5:**
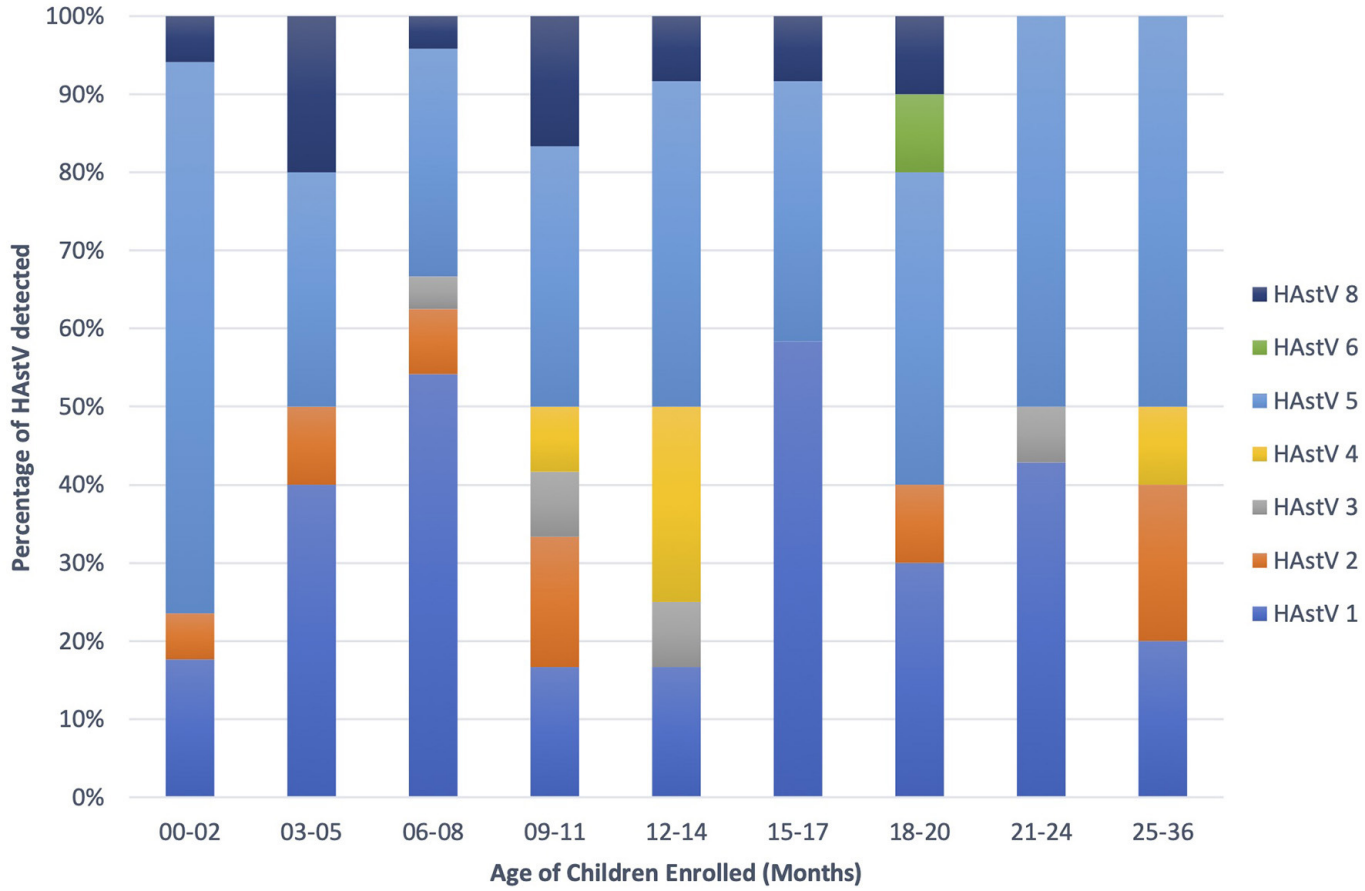
HAstV genotype distribution among age groups (0–36 months).

Astroviruses were detected throughout the year but were more frequent from December to February (Figure 4). Six different genotypes of human astrovirus were found in children between 6 and 14 months old, whereas 4 different astrovirus genotypes were seen among the children in the younger or older age groups. HAstV 1, HAstV 5, and HAstV 8 were seen in all age groups of children as shown in Figure 5.

### Phylogenetic Analysis of HAstV Strain

**Figure 6:**
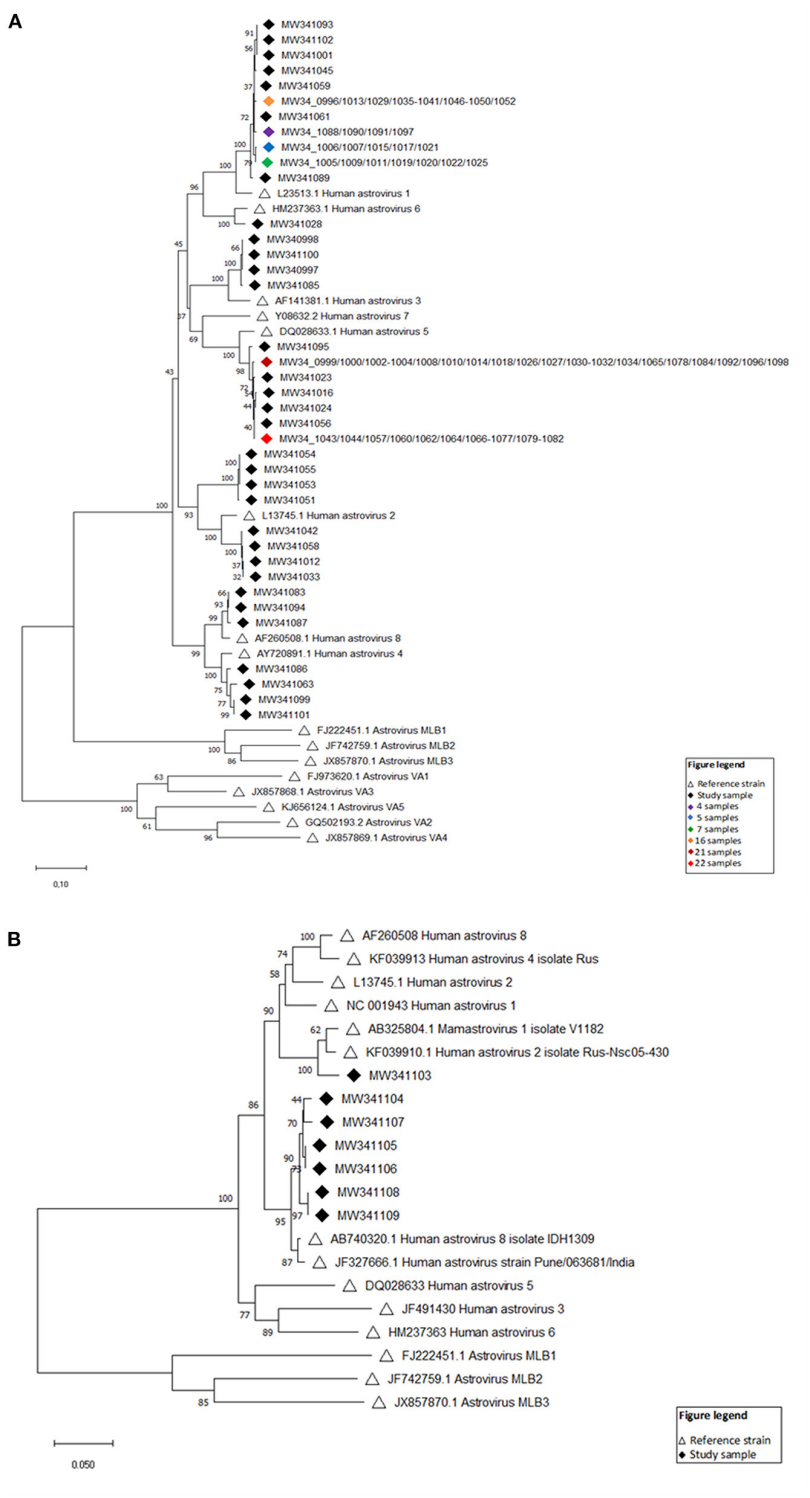
**(A)** Evolutionary relationships of taxa (ORF2) Neighbor-joining tree based on the ORF2 region. Sixteen reference strains were included in the phylogenetic analysis and are denoted by black open triangles with GenBank accession numbers indicated. Numbers at each node of the tree show bootstrap percentages obtained after 100 replicates. The viral strains identified in this study are listed by GenBank accession numbers with black diamonds, and groups of samples (>3) with identical strains are indicated by colored diamonds. **(B)** Evolutionary relationships of taxa (ORF1) Neighbor-joining tree based on the ORF1b region. Fourteen reference strains were included in the phylogenetic analysis and are denoted by black open triangles with GenBank accession numbers indicated. Numbers at each node of the tree show bootstrap percentages obtained after 100 replicates. The viral strains identified in this study are listed by GenBank accession numbers with black diamonds.

Phylogenetic analysis of nucleotide sequences from 114 HAstV (ORF1 and ORF2) fecal samples from infants and children showed large genetic variety among Nepalese children with all genotypes being detected except HAstV 7. Based on the ORF2 phylogeny, 39 specimens were HAstV 1, 8 were HAstV 2, 4 were HAstV 3, 4 were HAstV 4, 48 were HAstV 5, 1 was HAstV 6, and 3 were HAstV 8 (Figure 6A). The ORF1b phylogenetic tree additionally indicated that one more specimen was HAstV 1 while 6 fell in the HAstV 8 genotype (Figure 6B).

## Discussion

In this study, we described the prevalence, monthly distribution and the different strains of astroviruses circulating in cohort of children from birth till 36 months of age from a community in Bhaktapur, Nepal. In our study, astrovirus was found more in diarrheal stool samples than in non-diarrheal stool. Similarly, results from a birth cohort study completed in Vellore, India has also shown that astrovirus was associated with diarrhea (Holtz et al., 2011).

In this study, RT-PCR although it is a very sensitive assay, detected only 124 samples out of the 148 total positive samples by EIA. This could possibly be due to the presence of RT-PCR inhibitors present in fecal derived RNA or could be because of less PCR concentration (Lopez et al., 2017).

Like other studies from Ethiopia and Uruguay (Gelaw et al., 2019), we observed astrovirus infections in children older than 12 months old. Children <6 month of age were less likely to have astrovirus infections. This could explain the facts that at this age generally, infants are at home and are protected from direct contact with people and environment. Moreover, they will have breastfeed which in known to be protecting against viral and non-viral acute gastroenteritis (Lopez et al., 2017).

Astroviruses were detected throughout the year, but most frequently from December to February. Seasonality of astrovirus infections varies from one geographic region to another. United States, France, and Finland (Chikhi-Brachet et al., 2002) reported the occurrence of astrovirus in winter and spring whereas in Vietnam (Nguyen et al., 2008), it is reported in March to May.

Previous studies from Egypt, Italy, France, China, and Spain reported HAstV 1 as the predominant strain (Pang and Vesikari, 1999; Jeong et al., 2011). In countries like Korea, China and Japan HAstV 1 was reported in 70% of all HAstV infections, while Thailand reported 28%. In Mexico, HAstV 1 prevalence was low (10%) in comparison to HAstV 2 (42%), HAstV 4 (23%), HAstV 3 (13%), HAstV 5 (6%), and HAstV 7 (6%) in their study population (Walter and Mitchell, 2003). A study in Madagascar reported high prevalence of the HAstV 8 strain (Papaventsis et al., 2008). In contrast to all these studies, we found HAstV 5 as the most frequently detected genotype (42.2%), followed by HAstV 1 (34.7%), HAstV 2 and HAstV 8 (7.4%), HAstV 4 (4.1%), HAstV 3 (3.3%), and HAstV 6 (0.8%). However, predominant genotypes may change from one geographic region to another. In southeast Brazil, outbreaks associated with HAstV 2 have been reported among infants and children below 6 years of age (Gabbay et al., 2006) this is also reported in daycare centers in Virginia and hospitals in Melbourne, Australia. Similarly, infections with HAstV 3 were reported in Argentina, Wuhan in China and Thailand. The highly divergent human astrovirus MLB1 strain was identified from children with acute diarrhea in both Australia and USA. Novel strains (Ast-VA1) were also associated with an acute gastroenteritis outbreak reported from USA. Other novel human astrovirus closely related to mink and bovine astroviruses was found in human stool which indicates the zoonotic transmission. Novel astroviruses were not detected in our study, but to better understand genetic changes within the viral genome due to genetic evolution, it is important to analyze full genome sequences.

## Data Availability Statement

The raw data supporting the conclusions of this article will be made available by the authors, without undue reservation.

## Ethics Statement

The study was approved by ethical committee of Nepal Health Research Council (NHRC) and Regional Ethical Committee of Norway (REK42335).

## Author Contributions

All authors have contributed in planning the laboratory tests and preparing and reviewing the manuscripts.

## Conflict of Interest

The authors declare that the research was conducted in the absence of any commercial or financial relationships that could be construed as a potential conflict of interest.
